# Curcumin-Induced Global Profiling of Transcriptomes in Small Cell Lung Cancer Cells

**DOI:** 10.3389/fcell.2020.588299

**Published:** 2021-01-12

**Authors:** Fei Mo, Yinan Xiao, Hao Zeng, Dian Fan, Jinen Song, Xiaobei Liu, Meng Luo, Xuelei Ma

**Affiliations:** ^1^Department of Biotherapy, State Key Laboratory of Biotherapy, Cancer Center, West China Hospital, Sichuan University, Chengdu, China; ^2^Department of Medical Oncology, First Affiliated Hospital of Kunming Medical University, Kunming, China; ^3^Laboratory of Tumor Targeted and Immune Therapy, State Key Laboratory of Biotherapy, Clinical Research Center for Breast, West China Hospital, Sichuan University and Collaborative Innovation Center, Chengdu, China

**Keywords:** curcumin, small cell lung cancer, apoptosis, miR-548ah-5p, high-throughput sequencing

## Abstract

**Background:**

Curcumin, one of the promising candidates for supplementary therapy in cancer treatment, has been demonstrated by numerous preclinical and clinical evidence to be beneficial in treating various cancers. Apart from the critical role in a deluge of pathological processes, some mRNAs, in particular, microRNAs (miRNAs), are also involved in the anti-tumor activity. Therefore, our research focused on the possible effects of curcumin on small cell lung cancer (SCLC) cells and drew a comprehensive transcriptomes profile by high throughput sequencing to understand the molecular mechanism of curcumin as an anti-tumor agent.

**Methods:**

First, we calculated the apoptosis rate of H446 cells (a human SCLC cell line) cultured with curcumin. The high output sequencing uncovered the altered expression profile of genes and miRNAs. KEGG analysis selected the potential signal pathway associated with the antiproliferative property of curcumin. Finally, miRNAs significantly changed, as well as the regulatory roles of those miRNAs in cell apoptosis were determined.

**Result:**

The apoptosis rate of H446 cells increased under the elevated concentration of curcumin treatment. And cell cycle-related genes downregulated in the curcumin-treated cells. Besides, miRNA-548ah-5p of a high level acted as a negative role in the anticarcinogenic activity of curcumin.

**Conclusion:**

Our findings not only enriched the understanding of anti-tumor activity initiated by curcumin through figuring out the downregulated cell cycle-related pathways but also shed light on its novel therapeutic application.

## Introduction

Small cell lung cancer (SCLC) is one type of lung cancer, which accounts for less than 20% of all lung cancer cases diagnosed (Govindan et al., 2006). However, SCLC is more aggressive and has a greater tendency to spread to other organs in the body. Although advancements of chemotherapy and radiotherapy have improved the outcome of SCLC, its prognosis is still discouraging (Taniguchi et al., 2020). Therefore, more efforts are required to find new alternative strategies and enhance the effectiveness of integrated treatment strategies in SCLC.

Polyphenols, the most abundant organic compounds derived from a variety of plants and fruits, consist of various bioactive phytochemicals relevant to lower incidence of cardiovascular diseases, metabolic diseases, and cancer (Corrêa and Rogero, 2019). Given the innoxious effects on normal tissues and cells, previous studies focus on natural dietary polyphenols for their prominent roles in alleviating both side effects and multiple drug resistance induced by traditional anticarcinogens (Dei Cas and Ghidoni, 2018; Seca and Pinto, 2018). Recently, curcumin, a lipophilic flavonoid extracted from the rhizome of Curcuma longa, has been widely studied due to a spectrum of pharmacologic functions. Numerous preclinical and clinical trials have validated its role in a variety of human chronic diseases: inflammation, metabolic disorders, neurological, cardiovascular, infectious, skin diseases, and cancer (Kunnumakkara et al., 2017; Tomeh et al., 2019). The preventive and therapeutic roles of this bioactive polyphenolic compound might be attributable to its capability of modulating mRNA and miRNA expression profiles as well as the relevant signaling cascades, such as DNA methylation, histone modifications (Schnekenburger et al., 2019).

After firstly discovered in Caenorhabditis elegans in 1993, microRNAs, a group of evolutionarily conserved non-coding RNAs abundant in animals and plants, has long been confirmed to play a crucial role in a large number of biological and pathological processes (Lee et al., 1993). Binding to complementary sequences of targeted mRNA, usually, the 3’-untranslated region (3’-UTR), miRNAs are capable of silencing specific gene expression and ultimately forestalling translation of proteins at both transcriptional and post-transcriptional level, which consequently affect cell division, differentiation, and apoptosis (Miska, 2005; Pandima Devi et al., 2017). Functional studies have provided insights into roles of dysregulation of miRNAs attributable to genomic events in cancer and therefore implied the promise of novel miRNA-targeted chemotherapy (Rupaimoole and Slack, 2017).

Concerning 18.1 million newly reported cancer diagnoses and an estimated 9.6 million cancer death worldwide in 2018, cancer still aggravates the heavy global health burden (Bray et al., 2018). Among all malignant tumors, lung cancer turns out to be of the highest incidence rate and the leading cause of cancer mortality, which is subsequently followed by female breast cancer and prostate cancer (Bray et al., 2018). Owing to a better understanding of cancer biology, a paradigm shift in the cancer treatment field promotes the emergence of novel targeted chemotherapy. Since accumulating clinical evidence discloses that single tumor-targeted agent fails to generate reproducible therapeutic effects to patients because of the alternative activated signal pathway in tumor cells (Lopez and Banerji, 2017). Therefore, given complex biomolecular signal networks observed in tumor cells, combinations of targeted therapies are highly recommended to inhibit the activation of compensatory pathways.

Based on the aforementioned anti-tumor properties of curcumin, we aimed to uncover the critical intracellular processes initiated by curcumin in SCLC cells. We found that the cell cycle was significantly disrupted under curcumin treatment and confirmed that the miRNA-548ah-5p participated in the curcumin-induced cell apoptosis.

## Materials and Methods

### Chemical Reagents and Cell Culture

Curcumin was purchased from MedChem Express (MCE). Dimethyl sulfoxide (DMSO) was obtained from Sigma-Aldrich. The primers for miR-548ah-5p (#HmiRQP2080), miR-4725-3p (#HmiRQP2375), miR-195-5p (#HmiRQP0283), miR-10394-5p (#HmiRQP4666), and small nuclear RNA-U6 (RNU6, #HmiRQP9001) were provided by Genecopoeia. The negative control (NC) and inhibitors of miR-548ah-5p, miR-4725-3p, miR-195-5p, miR-10394-5p were purchased from GenePharma. The sequence of miR-548ah-5p inhibitor was: CAAACACUGCAAUCACUUUU. The sequence of miR-4725-3p inhibitor was: CCCGACACUGACGCCUUCCCCA. The sequence of miR-195-5p inhibitor was: GCCAAUAUUUCUGUGCUGCUA. The sequence of miR-10394-5p inhibitor was: AUGGCGUUCACCAGGACCUGCAGA. The sequence of inhibitor NC was: CAGUACUUUUGUGUAGUACAA. Curcumin was dissolved in DMSO at 50 μM to make a storage solution, then diluted with complete cell culture medium to various final concentrations. Cells were cultured in Dulbecco’s Modified Eagle Medium (DMEM), supplemented with 10% FBS, 100 U/mL penicillin and 100 μg/mL streptomycin in a humidified incubator containing 5% CO_2_ and 95% air at 37°C.

### Cell Proliferation Analysis

The effect of curcumin on cell proliferation was determined by Cell Counting Kit (CCK)-8 (MCE). First, H446 cells at logarithmic growth phase were seeded into the 96-wells plate at a density of 4 × 10^3^ cells/well. Subsequently, cells were treated with various concentrations of curcumin for 24 h. The cells in the curcumin (0 μM)-treated group were cultured with the same volume of DMSO as the curcumin (20 μM)-treated group. After incubation, CCK-8 kit assay was performed according to the manufacturer’s instruction. Briefly, 10 μL of CCK-8 solution was added to each well, and then the cells were maintained for 1 h at 37°C. The optical density (OD) was detected at 450 nm absorbance on the Microplate Reader (Bio-Rad).

### Apoptosis Assay

Annexin V-FITC/PI Detection Kit (BD Pharmingen) qualified the apoptosis rate of H446 cells after curcumin treatment. Cells were seeded into a 6-well plate overnight and left for adherence. After incubation with curcumin of various concentrations for 24 h, the adherent and detached cells were harvested and washed by PBS twice, then centrifuged at 1,200 rpm for 3 min. Subsequently, cells were resuspended in 100 μL of binding buffer. Then 5 μL of Annexin V-FITC and PI was added to the cell suspension and maintained for 15 min at room temperature in the dark. Finally, 400 μL of Binding Buffer was added and quantification of cell apoptosis was performed by NovoCyte flow cytometer (ACEABIO).

### TUNEL Assay

Terminal Deoxynucleotidyl Transferase-Mediated dUTP Nick-End Labeling (TUNEL) apoptosis assay kit (Beyotime) detected the apoptotic cells. In brief, cells were fixed with 4% paraformaldehyde, and washed with cold PBS three times. Next, the cells were permeabilized with 0.1% Triton X-100 in PBS. After washed three times, cells were stained with TUNEL detection reagent and counterstained with DAPI for 10 min. Finally, the fluorescence microscope (Leica DM2500) was used to visualize the positive-stained cells.

### Cell Cycle Analysis

H446 cells (1 × 10^5^ cell/well) were seeded in 6-well plates and cultured overnight. Then the cells were treated with curcumin (0, 5, 10 μM) for another 24 h. After harvested by trypsinization, cells were washed twice with PBS, then fixed with 70% ethanol overnight. Finally, they were stained by PBS containing 0.05 mg/mL PI, 1 μg/mL RNase, and 1 μg/mL Triton X-100 for 30 min. The fluorescence intensity of cells washed three times was quantified by flow cytometry.

### Western Blot Analysis

After curcumin treatment, total cellular proteins were extracted using RIPA lysis buffer that contains the protease inhibitor cocktail (Biosharp) on ice for 30 min. Then, the samples were centrifuged at 13,000 rpm for 15 min at 4°C to remove any cellular debris, collected, and quantified the supernatants using a bicinchoninic acid assay (BCA) protein assay kit (Thermo Fisher Scientific). Approximately 20 μg of protein samples loaded in each lane was used in sodium dodecyl sulfate-polyacrylamide gel electrophoresis (SDS-PAGE). The proteins were then transferred onto polyvinylidene fluoride (PVDF) membranes (Merck Millipore), followed by incubation in Tris-buffered saline with 0.1% Tween-20 (TBS-T) containing 5% skim milk (Biosharp) for 2 h to block non-specific protein binding. Next, the membranes were incubated with the following primary antibodies (diluted in TBS-T with 5% bovine serum albumin) at 4°C overnight: anti-Bcl-2, anti-Bax, anti-Cytochrome C and anti-β-actin (1:1,000 dilution). After washing three times with TBS-T, the membranes were incubated with secondary antibodies (Invitrogen) for 1 h at 37°C, and then washed with TBS-T three times. The protein bands of interest were visualized by enhanced chemiluminescence (ECL) method using SuperSignal West Pico Plus Chemiluminescent Substrate (Thermo Fisher Scientific).

### Next-Generation Sequencing (NGS) and Bioinformatic Analysis

Six samples of H446 cells, including three samples of the curcumin treatment group and three control samples, were prepared for the RNA sequencing process. The control group contained cells incubated with the same volume of DMSO as the curcumin (10 μM)-treated group did. Genes expressed in at least one sample were defined as detected genes. Differentially expressed genes (DEGs) referred to the genes meeting the criteria of | log2 (fold change)| > 1 with *p* < 0.05. The bioinformatic analysis further analyzed expression levels of microRNA, including the Kyoto Encyclopedia of Genes and Genomes (KEGG) enrichment analysis, Gene Ontology (GO) enrichment analysis, and heat map. KEGG pathway enrichment analysis could find the significant pathways^1^, and the criteria were count ≥ 3 and false discovery rate (FDR) < 0.05. GO functional enrichment analysis further assessed the biological process^2^, and the criteria were gene count ≥ 3 and *p* < 0.05.

### Quantitative Real Time-PCR Analysis

The total RNA was extracted from H446 cells by Buffer MZ (Tiangen) after incubation with or without curcumin at room temperature. The RNA was reversely transcribed to cDNA by MiRcute Plus miRNA First-Strand cDNA Synthesis kit (Tiangen). A quantitative real-time PCR was performed by MiRcute Plus miRNA qPCR kit (Tiangen). The primers of miR-548-5p, miR-4725-3p, miR-195-5p, miR-10394-5p, and small nuclear RNA-U6 (RNU6) were provided by Genecopoeia. The expression level of each microRNA was determined by miRNA sequence-specific primers and normalized to the expression level of RNU6.

### Transfection of MicroRNA Inhibitor

Cells were cultured in the growth medium for 24 h. Transfection of inhibitors of miR-548-5p, miR-4725-3p, miR-195-5p, miR-10394-5p, and control inhibitor was performed using Lipofectamine 3000 (Thermo Fisher Scientific) according to the manufacture’s protocol. After 24 h of transfection, cells were cultured in fresh medium with or without curcumin. And cells were collected to detect the cell proliferation and apoptosis using CCK-8 assay or FITC-annexin V/PI assay, respectively.

### Statistical Analysis

All data were presented as mean ± standard error (SE). The unpaired Student’s *t*-test analyzed the statistical difference between the two groups. A value of *p* < 0.05 was considered to indicate a statistical difference.

## Results

### Curcumin Reduced Cell Viability and Induced Cell Apoptosis in H446 Human SCLS Cell Line

**FIGURE 1:**
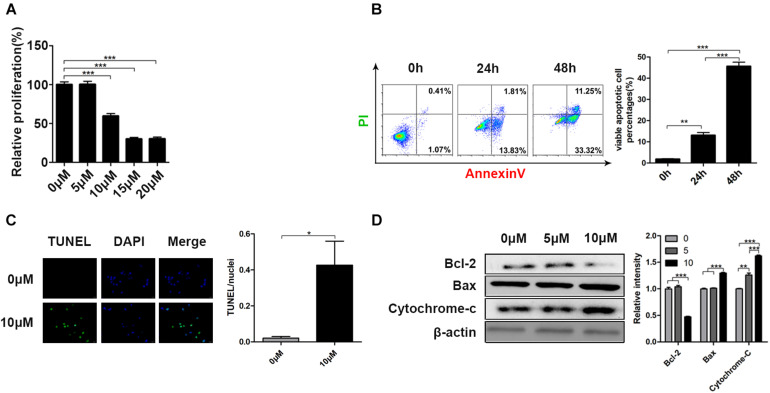
Curcumin reduced cell viability and induced cell apoptosis in H446 human SCLS cell line. **(A)** The relative proliferation of H446 cells was determined after 24 h curcumin treatment at different concentration (0, 5, 10, 15, 20 μM). **(B)** The apoptosis rate of H446 cells was evaluated by Annexin-V/PI apoptosis detection kit after 24 and 48 h curcumin (10 μM) treatment. The quantitative apoptotic percentages were shown. **(C)** After 24 h exposure to curcumin (10 μM), the apoptotic cells were identified by TUNEL apoptosis assay kit. **(D)** Expression of apoptosis-related proteins including Bcl-2, Bax, and cytochrome-C was detected after treatment of curcumin (0, 5, 10 μM) for 24 h. Data are showed as mean ± SEM (*n* = 3); **P < 0.01, ***P < 0.005.

To assess the anticarcinogenic property of curcumin, different cell lines (HCT116, Hela, MB231, PC-9, A549, H446) were cultured with curcumin. As shown in Supplementary Figure S1, the cell viability of these cell lines all decreased in a dose-dependent manner after curcumin treatment. Moreover, H466 cell lines were confirmed to be most sensitive to curcumin due to the most significant decline in cell viability than other cell types. Therefore, H446 cell lines were adopted for a subsequent experiment. And we obtained the cell viability of H446 cells under a lower concentration gradient after 24 h (Figure 1A). H446 cells were examined by Annexin-V/PI apoptosis detection kit after curcumin treatment at different time points to explore its effect of curcumin on cell apoptosis. As demonstrated in Figure 1B, the curcumin-induced cell apoptosis rate significantly increased from 3.03 to 12.82% or 46.82% at time points 24 or 48 h, respectively. Furthermore, the TUNEL assay indicated the apoptotic cells by marking DNA fragmentation (Figure 1C). As a result, TUNEL positive cells were detected in the curcumin-treated group. Besides, we examined the apoptosis-related protein expression after curcumin incubation (Figure 1D). The result showed that Curcumin treatment reduced the expression of Bcl-2 and increased the expression of Bax and cytochrome-C, which are involved in crucial regulation of cell growth (Ow et al., 2008). These results showed that curcumin effectively promoted the apoptosis of H446 cells.

### Transcriptome Alterations of H446 Cancer Cells Were Uncovered by High-Throughput Sequencing

**FIGURE 2:**
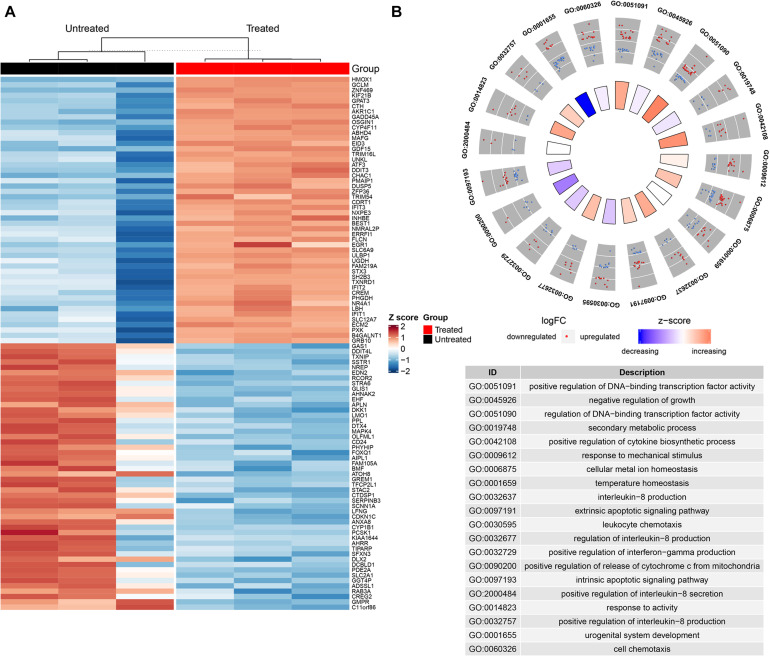
Transcriptome alterations of H446 cancer cells were uncovered by high-throughput sequencing. **(A)** The hierarchical clustering heatmap showed the significant differentiation of gene expression in H446 cells treated with or without curcumin (*n* = 3). The top 50 differentially expressed genes are provided on the right. Each column represented a tested sample and each row represented a given gene. The brightness of the color reflected the average expression level of certain gene, which had been standardized by *z*-score. The red indicated relatively higher gene expression while the blue represented lower gene expression. **(B)** GOPLOT of genes expressed differently (*p* < 0.05) in H446 cells treated with or without curcumin (*n* = 3) in biological process category. The red and blue spots in the scatter plot of out wheel showed upregulated and downregulated genes, respectively, while the inner wheel indicated the *z*-score.

A host of studies have unraveled that the curcumin suppressed cancer development via regulation of various biological molecules and activation of specific signal pathways (Giordano and Tommonaro, 2019). To explore the underlying mechanisms of curcumin, we utilized high throughput sequencing to identify the changes of mRNA and miRNA expression of H446 cells after curcumin treatment. Analysis of sequencing data between the curcumin-untreated or treated groups indicated a total of 21,660 expressed genes, 1,996 of which changed significantly (*p* < 0.05). Finally, 1,095 DEGs were selected by the DEseq2 (v 1.24.0) R package (Figure 2A), and related gene names were listed in Supplementary Table S1. Analysis of miRNA sequence data identified 1,566 miRNAs. Differential expression analysis further selected 70 miRNAs of significant expression difference (*p* < 0.05) between the two groups, including 38 up-regulated miRNAs and 32 down-regulated miRNAs. To determine the possible functions of enriched genes and signal pathways correlated with the anti-neoplasm property of curcumin, gene ontology (GO) enrichment analysis was applied to explore the role of DEGs under curcumin treatment (Figure 2B). The result demonstrated that the most enriched terms were relevant to the biological process on the regulation of DNA-binding transcription factor activity.

### MiR-548ah-5p Regulated the Curcumin-Induced Apoptosis of H446 Cell Line

**FIGURE 3:**
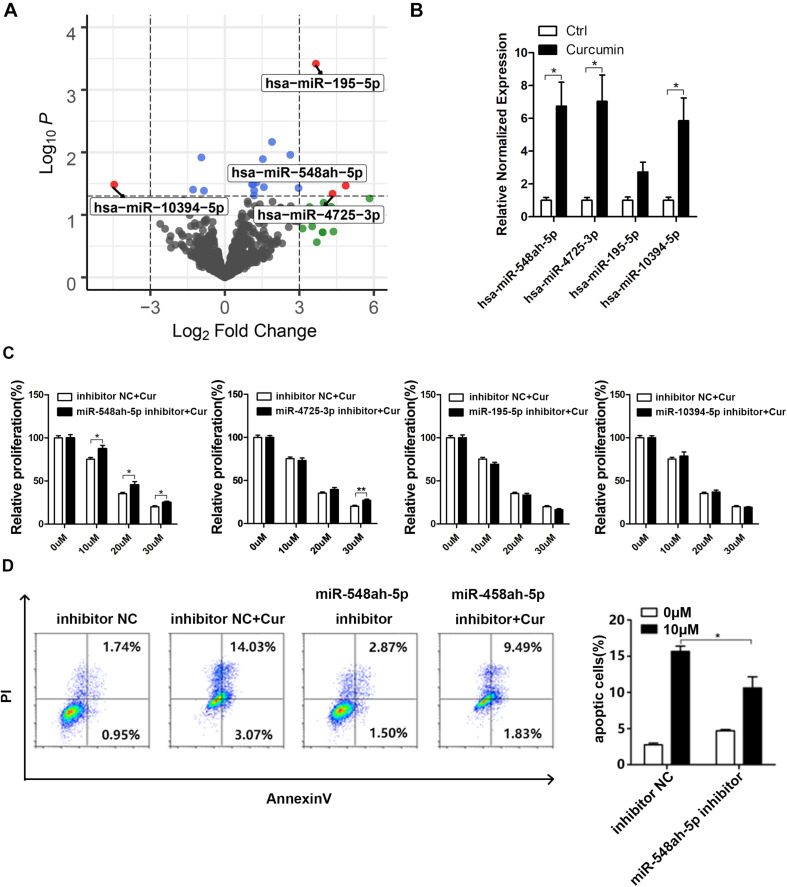
MiR-548ah-5p regulated the curcumin-induced apoptosis of H446 cell line. **(A)** Volcano plot of all miRNA based on log2 fold change vs. the -log10 *p*-value. Each dot represented a miRNA, and significantly differentially expressed miRNAs were labeled as red. **(B)** Relative expression levels of miR-548ah-5p, miR-4725-3p, miR-195-5p, and miR-10394-5p were determined in H446 cells pre-treated with 10 μM curcumin. **(C)** Relative viability of H446 cells pre-transfected with the inhibitor of miR-548ah-5p, miR-4725-3p, miR-195-5p, or miR-10394-5p was determined by CCK-8 assay after 24 h curcumin treatment (0, 10, 20, 30 μM). **(D)** H446 cells transfected with the inhibitor of miR-548ah-5p were treated with curcumin (10 μM) for 24 h and then the apoptotic cells were identified by Apoptosis Assays Kit. Quantification of apoptosis rate were shown. Data are showed as mean ± SEM (*n* = 3); *P < 0.05.

Based on the bioinformatic analysis, four miRNAs, including miR-548ah-5p, miR-4725-3p, miR-195-5p, and miR-10394-5p, were found to be the most significantly changed miRNAs in the curcumin-treated group (Figure 3A). To prove the reliability of the bioinformatic result, we extracted the total miRNA of curcumin-treated H446 cells and quantified the relative normalized expression of these miRNAs by qRT-PCR (Figure 3B). All the tested miRNAs except miR-195-5p showed a significant elevation compared with the control. However, the regulatory effect of these miRNAs on the cell apoptosis remained to be unknown. Therefore, the H446 cancer cells were transfected with the specific inhibitors of these miRNAs and treated with curcumin at different concentrations (Figure 3C). As a result, both miR-548ah-5p and miR-4725-3p inhibitors weakened the negative influence of curcumin on cell viability at different concentrations (10, 20, 30 μm). To further confirm the regulatory role of miR-548ah-5p and miR-4725-3p in curcumin-induced apoptosis, expressions of miR-548ah-5p and miR-4725-3p were pre-refrained by specific inhibitors in H446 cells and determined the rates of cell apoptosis after curcumin incubation (Figure 3D). The result showed that the apoptotic cells decreased sharply after the transfection of the miRNA-548ah-5p inhibitor. However, there was no significant difference in cell apoptosis in miRNA-4725-3p inhibitor-transfected group. These results above validated the positive effect of miR-548ah-5p in curcumin-induced cell apoptosis.

### The Disturbation of Cell Cycle-Related Pathways Contributed to the Cell Apoptosis

**FIGURE 4:**
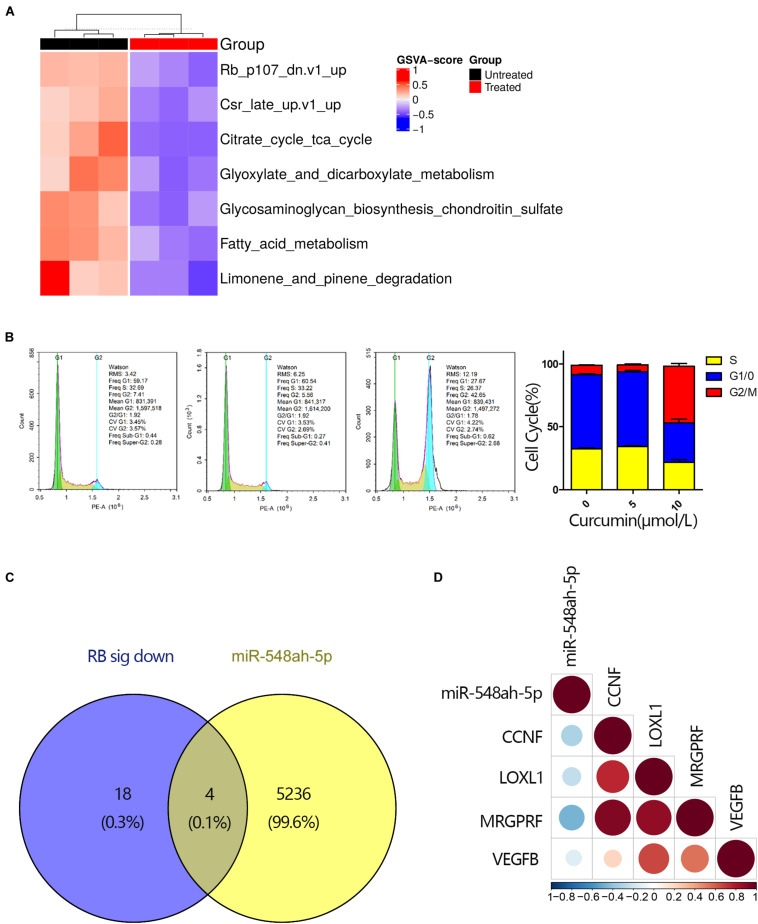
The disturbation of cell cycle-related pathways contributed to the cell apoptosis. **(A)** GSVA score for differential KEGG gene sets between curcumin-untreated and treated groups (*n* = 3). Each column represented a tested sample while each row represented a KEGG pathway. GSVA scores reflected the variation in activeness of different pathways. Hierarchical clustering heatmap indicated differential oncogenic gene sets and the brightness of the color was related to GSVA scores. **(B)** Cell cycle analysis for H446 cells was measured by flow cytometry after cultured with curcumin (0, 5, 10 μM) for 24 h and ratio of different phases were shown (*n* = 3). **(C)** Venn diagram of all down-regulated DEGs in the RB signature and genes targeted by miRNA-548ah-5p. **(D)** The relevance between miRNA-548ah-5p expression and its potential target genes in RB signature. Colors and sizes of the spots represent the relevance scores.

To determine the mechanism of cell apoptosis induced by curcumin, we conducted the gene set enrichment analysis by GSVA approach (Figure 4A). Cell cycle-related gene sets significantly changed under curcumin treatment, especially the RB gene set. As shown in Figrue 4B, cell cycle analysis indicated that the G2/M population ratio significantly increased in the 10 μM curcumin-treated group compared with other groups. Since high expression levels of miR-548ah-5p, as well as downregulation of the RB gene set, were found in the apoptosis of H446 cell lines under the treatment of curcumin, we wondered whether the miR-548ah-5p interacted with the genes in RB gene sets to play a regulatory role in the cell cycle. Therefore, we selected the intersection of 5,243 miR-548ah-5p-target genes and all down-regulated genes contained in the RB gene set signatures in Figure 4C. The result showed that only four genes, CCNF, LOX1, MRGPRF, and VEGFB, remained in the intersection (Figure 4C). All of them had a negative correlation with miRNA-548ah-5p (Figure 4D). These data suggested that curcumin promoted cell apoptosis via cell cycle arrest. Moreover, miRNA-548ah-5p might involve in the regulation of cell cycle-related gene expressions according to the bioinformatics analysis.

## Discussion

Accumulating studies have demonstrated that curcumin is capable of inhibiting tumor cell growth and suppressing metastasis via regulation of various biomolecules such as adhesion molecules, cytokines, growth factors, and their receptors (Bachmeier et al., 2018). Curcumin inhibits the proliferation, metastasis, invasion and angiogenesis process of non-SCLC cells by regulation of the tumor-related genes and downstream signaling pathways (Shishodia, 2013; Imran et al., 2018). Previous studies have demonstrated that the suppression of STAT3 phosphorylation and VEGF expression, as well as inhibition of MMP-2 and MMP-9, contributes to alleviating the invasion and angiogenesis process of lung cancer cells by curcumin (Lin et al., 2009; Yang et al., 2012). However, few studies indicates the role of curcumin in SCLC cells. In our experiment, we found that the apoptosis rate of H446 cell lines was elevated as the dose of curcumin increased. We further explored the possible mechanisms by methods of bioinformatics, which may provide new clues to support its fascinating anti-tumor properties.

Notably, given the positive role of curcumin in suppressing carcinogenesis *in vivo* and *in vitro*, curcumin is expected to be a potential anticarcinogenic agent (Xiang et al., 2020). However, the potential application of curcumin might face a great barrier: the low bioavailability, mainly owing to rapid systemic elimination and poor water solubility (Barati et al., 2019). Fortunately, as recently stated, the improvement of nanotechnology permits the encapsulated drug delivery, which could make up for these disadvantages mentioned above and the side effects of curcumin (Panahi et al., 2019). Additionally, curcumin, the chemo-sensitive agents, when combined with chemical anti-cancer agents, possesses multiple properties, including reversing multidrug-resistance, minimalizing the dosage and optimizing its efficacy (Batra et al., 2019).

As a potential anticarcinogen, curcumin could regulate NF-κB (Zhang et al., 2013), PI3K/Akt (Jin et al., 2015), and Wnt/β-catenin (Lu et al., 2014) to impose proapoptotic effects on non-SCLC cells. However, there are few studies on the effect and relative molecular mechanisms of curcumin in SCLC. Here, we determined all statistically altered signaling pathways in SCLC cells induced by curcumin with bioinformatics analysis. Notably, we found that the gene sets associated with advancing the cell cycle process, especially RB gene set, were significantly downregulated in H446 cells under curcumin treatment. RB signature is a set of genes up-regulated in primary keratinocytes from RB1 and RBL1 skin-specific knockout mice, which leads to increased cell proliferation, aberrant differentiation and the disengagement of these processes (Saiz-Ladera et al., 2014). In line with the discovery, cell cycle analysis confirmed the G2/M cycle arrest of H446 cells with curcumin treatment.

As a potential anti-cancer agent, curcumin is capable of inhibiting cancer development via the miRNA-mediated mechanism (Momtazi et al., 2016). MiRNAs, a family of endogenous non-decoding RNAs, silences the specific genes by facilitating mRNA degeneration in the form of RNA-induced silencing complex at the transcriptional and post-transcriptional level (Pandima Devi et al., 2017). Even though the comprehensive functions of the miRNA family in various human diseases have not been clarified yet (Pavan et al., 2016), more researches sheds light on the contributions of specific miRNAs to mitigating some malignant diseases. Acted as crucial regulators involved in the function of curcumin, miRNA-186 (Zhang et al., 2010), miR-21 (Zhang et al., 2014), miRNA-98 (Liu et al., 2017), miRNA-192-5p, and miR-215 (Ye et al., 2015) are reported to be responsible for restricting initiation and progression of cancer by targeting relative molecules and downstream signaling pathways. In our study, we observed that the curcumin-induced cell apoptosis was along with the elevated expression of miRNA-548ah-5p detected by transcriptome sequencing. miRNA-548ah-5p is reported to participate in the regulation of chronic hepatitis B (Xing et al., 2014). To further determine the relevance of curcumin-induced apoptosis and miRNA-548ah-5p, H446 cells were transfected with miRNA-548ah-5p inhibitor, then measured the percentage of apoptosis cells under the treatment of curcumin. At a limited level of miRNA-548ah-5p, the relative proliferation of H446 cell lines rebounded, which indicated that the efficiency of curcumin partly relied on increasing the expression of miRNA-548ah-5p. Besides, the correlation between miR-548ah-5p and the down-regulated genes in RB signatures suggested that the miR-548ah-5p might participate in regulating the expression of genes involved in the process of H446 cell cycle.

## Conclusion

Overall, in our study, the apoptosis and restricted viability in H446 SCLC cells induced by curcumin showed the therapeutic properties of the herbal extract. The comprehensive analysis of changed mRNAs and miRNAs made up for the blanks of the complicated regulatory mechanism of curcumin-induced apoptosis of SCLC cells. Moreover, miRNA-548ah-5p may regulate the expression of genes involved in the process of cell cycle to promote SCLC cell apoptosis, which further requires more study to confirm its role in anti-cancer efficiency. Besides, more animal and clinical trials are necessary to identify the therapeutic value of curcumin in SCLC.

## Data Availability Statement

The raw data supporting the conclusions of this article will be made available by the authors, without undue reservation.

## Author Contributions

FM and YX wrote and modified the manuscript. FM, HZ, DF, ML, and XL collected the samples and performed the related experiments. JS analyzed the data. XM designed this project. All authors listed have made a substantial, direct and intellectual contribution to the work, and approved it for publication.

## Conflict of Interest

The authors declare that the research was conducted in the absence of any commercial or financial relationships that could be construed as a potential conflict of interest.

## Footnotes

1 http://www.genome.jp/kegg/

2 http://www.geneontology.org

## References

[B1] Bachmeier B. E. Killian P. H. Melchart D. (2018). The role of curcumin in prevention and management of metastatic disease. *Int. J. Mol. Sci.* 19:1716. 10.3390/ijms19061716 PMC6032261 29890744

[B2] Barati N. Momtazi-Borojeni A. A. Majeed M. Sahebkar A. (2019). Potential therapeutic effects of curcumin in gastric cancer. *J. Cell. Physiol.* 234 2317–2328. 30191991 10.1002/jcp.27229

[B3] Batra H. Pawar S. Bahl D. (2019). Curcumin in combination with anti-cancer drugs: a nanomedicine review. *Pharmacol. Res.* 139 91–105. 30408575 10.1016/j.phrs.2018.11.005

[B4] Bray F. Ferlay J. Soerjomataram I. Siegel R. L. Torre L. A. Jemal A. (2018). Global cancer statistics 2018: GLOBOCAN estimates of incidence and mortality worldwide for 36 cancers in 185 countries. *CA Cancer J. Clin.* 68 394–424. 30207593 10.3322/caac.21492

[B5] Corrêa T. A. Rogero M. M. (2019). Polyphenols regulating microRNAs and inflammation biomarkers in obesity. *Nutrition* 59 150–157. 30471527 10.1016/j.nut.2018.08.010

[B6] Dei Cas M. Ghidoni R. (2018). Cancer prevention and therapy with polyphenols: sphingolipid-mediated mechanisms. *Nutrients* 10:940. 10.3390/nu10070940 PMC6073226 30037082

[B7] Giordano A. Tommonaro G. (2019). Curcumin and cancer. *Nutrients* 11:2376. 10.3390/nu11102376 PMC6835707 31590362

[B8] Govindan R. Page N. Morgensztern D. Read W. Tierney R. Vlahiotis A. (2006). Changing epidemiology of small-cell lung cancer in the United States over the last 30 years: analysis of the surveillance, epidemiologic, and end results database. *J. Clin. Oncol.* 24 4539–4544. 17008692 10.1200/JCO.2005.04.4859

[B9] Imran M. Ullah A. Saeed F. Nadeem M. Arshad M. U. Suleria H. A. R. (2018). Cucurmin, anticancer, & antitumor perspectives: a comprehensive review. *Crit. Rev. Food Sci. Nutr.* 58 1271–1293. 27874279 10.1080/10408398.2016.1252711

[B10] Jin H. Qiao F. Wang Y. Xu Y. Shang Y. (2015). Curcumin inhibits cell proliferation and induces apoptosis of human non-small cell lung cancer cells through the upregulation of miR-192-5p and suppression of PI3K/Akt signaling pathway. *Oncol. Rep.* 34 2782–2789. 26351877 10.3892/or.2015.4258

[B11] Kunnumakkara A. B. Bordoloi D. Padmavathi G. Monisha J. Roy N. K. Prasad S. (2017). Curcumin, the golden nutraceutical: multitargeting for multiple chronic diseases. *Br. J. Pharmacol.* 174 1325–1348. 27638428 10.1111/bph.13621 PMC5429333

[B12] Lee R. C. Feinbaum R. L. Ambros V. (1993). The C. elegans heterochronic gene lin-4 encodes small RNAs with antisense complementarity to lin-14. *Cell* 75 843–854. 8252621 10.1016/0092-8674(93)90529-y

[B13] Lin S. S. Lai K. C. Hsu S. C. Yang J. S. Kuo C. L. Lin J. P. (2009). Curcumin inhibits the migration and invasion of human A549 lung cancer cells through the inhibition of matrix metalloproteinase-2 and -9 and vascular endothelial growth factor (VEGF). *Cancer Lett.* 285 127–133. 19477063 10.1016/j.canlet.2009.04.037

[B14] Liu W. L. Chang J. M. Chong I. W. Hung Y. L. Chen Y. H. Huang W. T. (2017). Curcumin inhibits LIN-28A through the activation of miRNA-98 in the lung cancer cell line A549. *Molecules* 22:929. 10.3390/molecules22060929 PMC6152786 28587210

[B15] Lopez J. S. Banerji U. (2017). Combine and conquer: challenges for targeted therapy combinations in early phase trials. *Nat. Rev. Clin. Oncol.* 14 57–66. 27377132 10.1038/nrclinonc.2016.96 PMC6135233

[B16] Lu Y. Wei C. Xi Z. (2014). Curcumin suppresses proliferation and invasion in non-small cell lung cancer by modulation of MTA1-mediated Wnt/β-catenin pathway. *In Vitro Cell. Dev. Biol. Anim.* 50 840–850. 24938356 10.1007/s11626-014-9779-5

[B17] Miska E. A. (2005). How microRNAs control cell division, differentiation and death. *Curr. Opin. Genet. Dev.* 15 563–568. 16099643 10.1016/j.gde.2005.08.005

[B18] Momtazi A. A. Shahabipour F. Khatibi S. Johnston T. P. Pirro M. Sahebkar A. (2016). Curcumin as a MicroRNA regulator in cancer: a review. *Rev. Physiol. Biochem. Pharmacol.* 171 1–38. 27457236 10.1007/112_2016_3

[B19] Ow Y. P. Green D. R. Hao Z. Mak T. W. (2008). Cytochrome c: functions beyond respiration. *Nat. Rev. Mol. Cell Biol.* 9 532–542. 18568041 10.1038/nrm2434

[B20] Panahi Y. Fazlolahzadeh O. Atkin S. L. Majeed M. Butler A. E. Johnston T. P. (2019). Evidence of curcumin and curcumin analogue effects in skin diseases: a narrative review. *J. Cell. Physiol.* 234 1165–1178. 30073647 10.1002/jcp.27096

[B21] Pandima Devi K. Rajavel T. Daglia M. Nabavi S. F. Bishayee A. Nabavi S. M. (2017). Targeting miRNAs by polyphenols: novel therapeutic strategy for cancer. *Semin. Cancer Biol.* 46 146–157. 28185862 10.1016/j.semcancer.2017.02.001

[B22] Pavan A. R. Silva G. D. Jornada D. H. Chiba D. E. Fernandes G. F. Man Chin C. (2016). Unraveling the anticancer effect of curcumin and resveratrol. *Nutrients* 8:628. 10.3390/nu8110628 PMC5133053 27834913

[B23] Rupaimoole R. Slack F. J. (2017). MicroRNA therapeutics: towards a new era for the management of cancer and other diseases. *Nat. Rev. Drug Discov.* 16 203–222. 28209991 10.1038/nrd.2016.246

[B24] Saiz-Ladera C. Lara M. F. Garin M. Ruiz S. Santos M. Lorz C. (2014). p21 suppresses inflammation and tumorigenesis on pRB-deficient stratified epithelia. *Oncogene* 33 4599–4612. 24121270 10.1038/onc.2013.417 PMC4913869

[B25] Schnekenburger M. Dicato M. Diederich M. F. (2019). Anticancer potential of naturally occurring immunoepigenetic modulators: a promising avenue? *Cancer* 125 1612–1628. 30840315 10.1002/cncr.32041

[B26] Seca A. M. L. Pinto D. (2018). Plant secondary metabolites as anticancer agents: successes in clinical trials and therapeutic application. *Int. J. Mol. Sci.* 19:263. 10.3390/ijms19010263 PMC5796209 29337925

[B27] Shishodia S. (2013). Molecular mechanisms of curcumin action: gene expression. *BioFactors* 39 37–55. 22996381 10.1002/biof.1041

[B28] Taniguchi H. Sen T. Rudin C. M. (2020). Targeted therapies and biomarkers in small cell lung cancer. *Front. Oncol.* 10:741. 10.3389/fonc.2020.00741 32509576 PMC7251180

[B29] Tomeh M. A. Hadianamrei R. Zhao X. (2019). A review of curcumin and its derivatives as anticancer agents. *Int. J. Mol. Sci.* 20:1033. 10.3390/ijms20051033 PMC6429287 30818786

[B30] Xiang D. B. Zhang K. Q. Zeng Y. L. Yan Q. Z. Shi Z. Tuo Q. H. (2020). Curcumin: from a controversial “panacea” to effective antineoplastic products. *Medicine* 99:e18467. 10.1097/MD.0000000000018467 PMC6959860 31914018

[B31] Xing T. J. Xu H. T. Yu W. Q. Wang B. Zhang J. (2014). MiRNA-548ah, a potential molecule associated with transition from immune tolerance to immune activation of chronic hepatitis B. *Int. J. Mol. Sci.* 15 14411–14426. 25196343 10.3390/ijms150814411 PMC4159859

[B32] Yang C. L. Liu Y. Y. Ma Y. G. Xue Y. X. Liu D. G. Ren Y. (2012). Curcumin blocks small cell lung cancer cells migration, invasion, angiogenesis, cell cycle and neoplasia through Janus kinase-STAT3 signalling pathway. *PLoS One* 7:e37960. 10.1371/journal.pone.0037960 22662257 PMC3360669

[B33] Ye M. Zhang J. Zhang J. Miao Q. Yao L. Zhang J. (2015). Curcumin promotes apoptosis by activating the p53-miR-192-5p/215-XIAP pathway in non-small cell lung cancer. *Cancer Lett.* 357 196–205. 25444916 10.1016/j.canlet.2014.11.028

[B34] Zhang B. Y. Shi Y. Q. Chen X. Dai J. Jiang Z. F. Li N. (2013). Protective effect of curcumin against formaldehyde-induced genotoxicity in A549 cell lines. *J. Appl. Toxicol.* 33 1468–1473. 23059809 10.1002/jat.2814

[B35] Zhang J. Du Y. Wu C. Ren X. Ti X. Shi J. (2010). Curcumin promotes apoptosis in human lung adenocarcinoma cells through miR-186^∗^ signaling pathway. *Oncol. Rep.* 24 1217–1223. 20878113 10.3892/or_00000975

[B36] Zhang W. Bai W. Zhang W. (2014). MiR-21 suppresses the anticancer activities of curcumin by targeting PTEN gene in human non-small cell lung cancer A549 cells. *Clin. Transl. Oncol.* 16 708–713. 24293118 10.1007/s12094-013-1135-9

