# Supplementary figures and images for: Curcumin-Induced Global Profiling of Transcriptomes in Small Cell Lung Cancer Cells
Source: Front Cell Dev Biol. 2021 Jan 12;8:588299. doi: 10.3389/fcell.2020.588299 (PMC7835540; doi:10.3389/fcell.2020.588299)

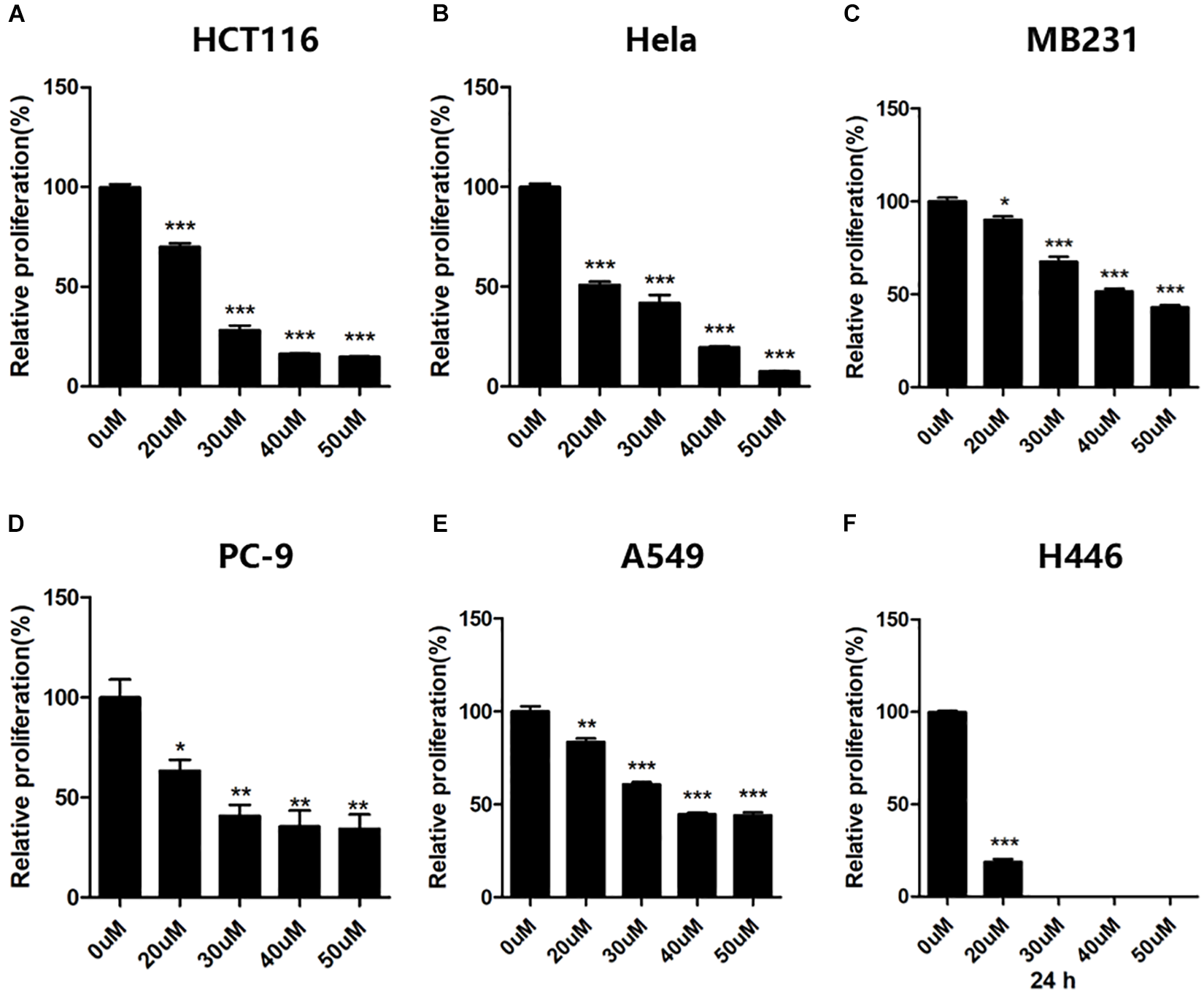

Supplement: Supplementary file 1 [file Image_1.tif]

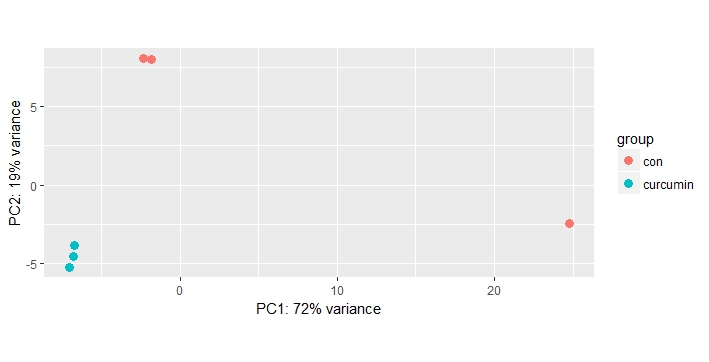

Supplement: Supplementary file 3 [file Data_Sheet_2.ZIP › cur mrna pca.jpeg]

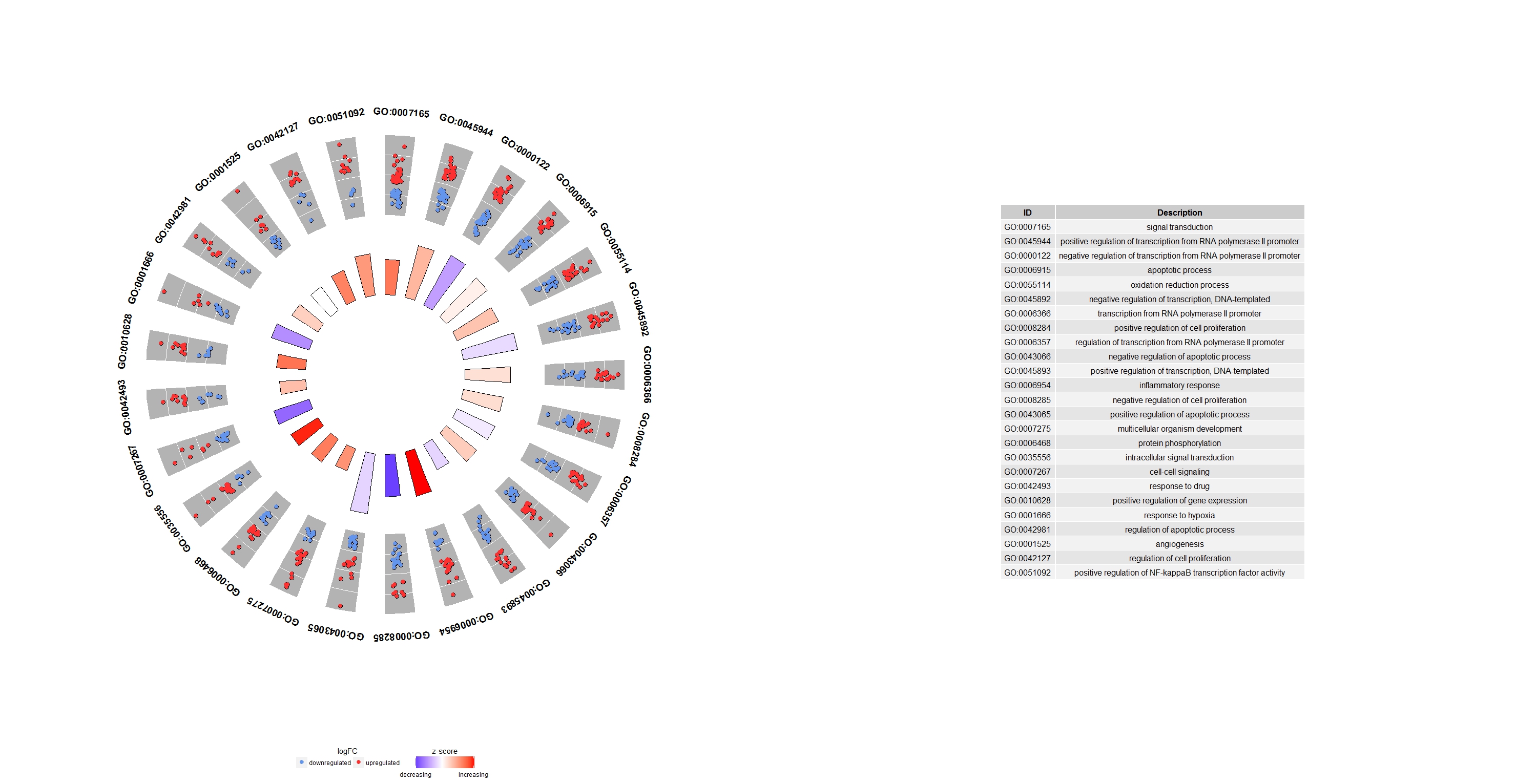

Supplement: Supplementary file 3 [file Data_Sheet_2.ZIP › go.jpeg]

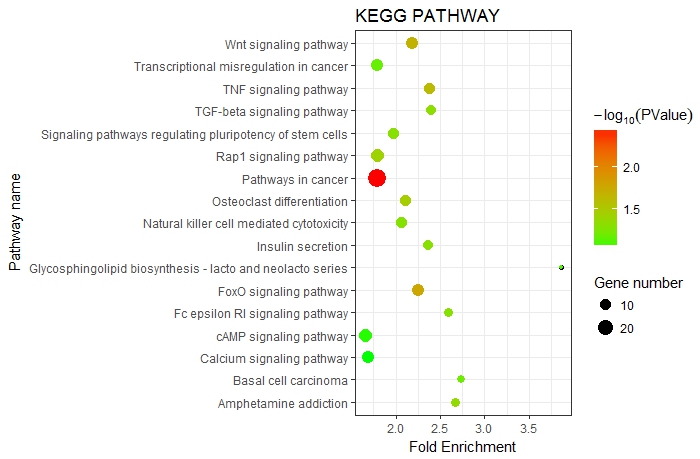

Supplement: Supplementary file 3 [file Data_Sheet_2.ZIP › kegg.jpeg]

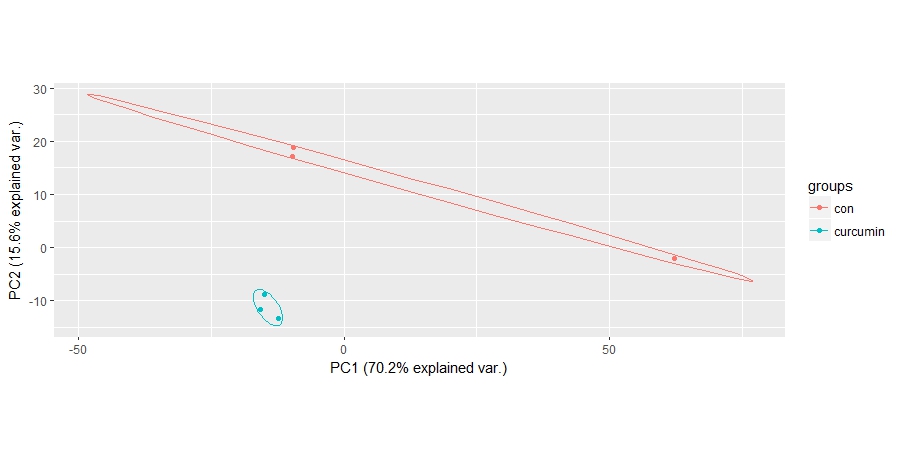

Supplement: Supplementary file 3 [file Data_Sheet_2.ZIP › pca mrna 2 cur.jpeg]
